# Application of “Click” Chemistry in Biomedical Hydrogels

**DOI:** 10.1021/acsomega.2c03931

**Published:** 2022-10-12

**Authors:** Xin Li, Yuzhu Xiong

**Affiliations:** Department of Polymer Materials and Engineering, Guizhou University, Guiyang 550025, P. R. China

## Abstract

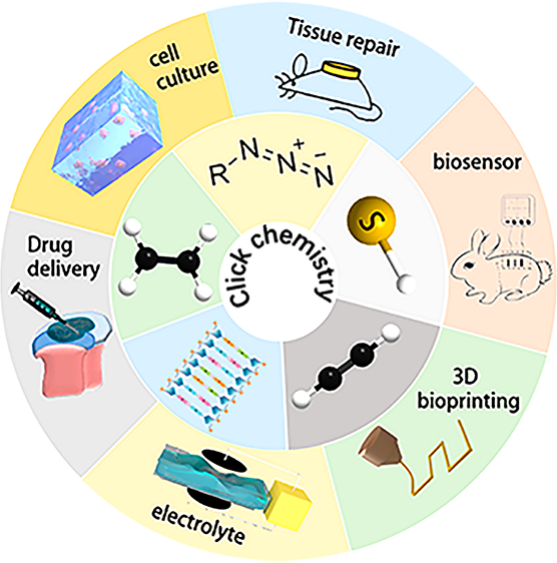

Since “click” chemistry was first reported in 2001, it has remained a popular research topic in the field of chemistry due to its high yield without byproducts, fast reaction rate, simple reaction, and biocompatibility. It has achieved good applications in various fields, especially for the preparation of hydrogels. The development of biomedicine presents new challenges and opportunities for hydrogels, and “click” chemistry provides a library of chemical tools for the preparation of various innovative hydrogels, including cell culture, 3D bioprinting, and drug release. This article summarizes several common “click” reactions, including copper-catalyzed azide–alkyne cycloaddition reactions, strain-promoted azide–alkyne cycloaddition (SPAAC) reaction, thiol–ene reaction, the Diels–Alder reaction, and the inverse electron demand Diels–Alder (IEDDA) reaction. We introduce the “click” reaction in the nucleic acid field to expand the concept of “click” chemistry. This article focuses on the application of “click” chemistry for preparing various types of biomedical hydrogels and highlights the advantages of “click” reactions for cross-linking to obtain hydrogels. This review also discusses applications of “click” chemistry outside the field of hydrogels, such as drug synthesis, targeted delivery, and surface modification, hydrogels have great application potential in these fields in the future and hopefully inspire other applications of hydrogels.

## 1 Introduction

Hydrogels are polymeric materials with a 3D network structure with a water content of up to 99%. They have a wide range of applications in various disciplines due to their unique properties and functionalities,^1−3^ especially in the field of biomedicine.^4−8^ Hydrogels have high water retention and good mechanical properties, similar to human soft tissues, and have great potential biomedical applications,^9^ such as drug delivery,^10^ wound healing,^11^ and tissue repair.^12^ In addition to the biological field, many other fields have shown a fondness for hydrogels, which have jointly contributed to the development of hydrogels by exploring and improving the polymer and cross-linking reactions of hydrogels.^13^ Hydrogels can be cross-linked either physically or chemically.^14^ Physically cross-linked hydrogels are mainly connected by noncovalent bonds, such as ionic bonds, hydrogen bonds, and molecular entanglements.^15^ Chemically cross-linked hydrogels are mainly connected by covalent bonds, which are stronger than physical cross-links. The main cross-linking mechanisms include free-radical polymerization, “click” chemistry cross-linking, and enzyme-induced cross-linking.^16^ Compared with physical cross-linking, chemically cross-linked hydrogel networks have better mechanical properties, greater stability, and better applications in various fields.^17^ A generally applicable cross-linking method has been explored for nearly a century. The system is very mature, and it is difficult to seek breakthroughs in technology. “Click” chemistry was discovered very late, and it is in the stage of gradual improvement. It has high research value, a fast reaction rate, and a high selectivity, giving it unique advantages for hydrogel cross-linking. Therefore, it has attracted significant attention for the synthesis of hydrogels and has become one of the most widely used synthetic methods.

“Click” chemistry is a spontaneous, rapid, highly selective, and high-yield chemical reaction between two molecules under mild conditions.^18^ Taking a hydrogel as an example, there are no byproducts during its synthesis, or the only byproduct is water.^19^ Since its discovery, “click” chemistry has promoted the development of many functional substances and new materials with applications in medicine,^20^ agriculture,^21−23^ and other fields. In the field of hydrogels, “click” chemistry hydrogels are now gaining popularity due to the development of their powerful selectivity and bioorthogonal reactions in physiological environments.^24^ The use of “click” chemistry allows multiple strategies to cross-link hydrogels and tailor their physical and chemical properties. The first to use “click” chemistry to prepare hydrogels was Hubbell and colleagues,^25^ who used Michael addition to prepare biohydrogels for tissue repair. Since then, many other scientists have devoted themselves to the development of “click” chemistry for hydrogels in various fields.

In this review, we focus on hydrogels cross-linked by “click” chemistry and their applications in biomedical fields. First, we introduce several common “click” chemistries. To expand our understanding of “click” chemistry, we also introduce “click” chemistry in the field of nucleic acid as a supplement. Then we discuss hydrogels cross-linked using “click” chemistry and review the current state of the application of “click chemistry” in biomedical hydrogels, highlighting their advantages over other cross-linking methods. In addition to the application of “click” chemistry in biomedical hydrogels, we also introduce the application of “click” chemistry in areas where hydrogels have potential applications in the future.^26^ This review also provides new insights into the design and applications of hydrogel materials in the future to provide some new ideas for researchers in various fields.

## 2 Classification of “Click” Chemistry

“Click” chemistry is a concept proposed by Sharpless and colleagues in 2001.^27^ Its principle was quickly adopted, and it was a breakthrough in synthetic chemistry, inspiring scientists in almost all fields of chemistry.^28^ “Click” chemistry has a fast reaction rate, high selectivity, simple reaction conditions, and almost no byproducts.^29^ It has been widely used in the field of hydrogel synthesis. “Click” chemistry not only expands the synthesis methods of hydrogels to obtain desired structures and properties but also facilitates access to hydrogels for those outside the field of chemistry.^24^ “Click” chemistry is not a specific reaction, but rather a general term for many reactions with the same characteristics. In this section, we will introduce several important “click” reactions, such as the copper-catalyzed CuAAC reaction^30^ and thiol–ene reactions between thiols and double bonds.^31^ We will also introduce some special “click” reactions to provide a more intuitive and comprehensive understanding of “click” chemistry.

### 2.1 Copper-Ion-Catalyzed Azide–Alkyne Cycloaddition Reaction (CuAAC Reaction)

**Figure 1:**
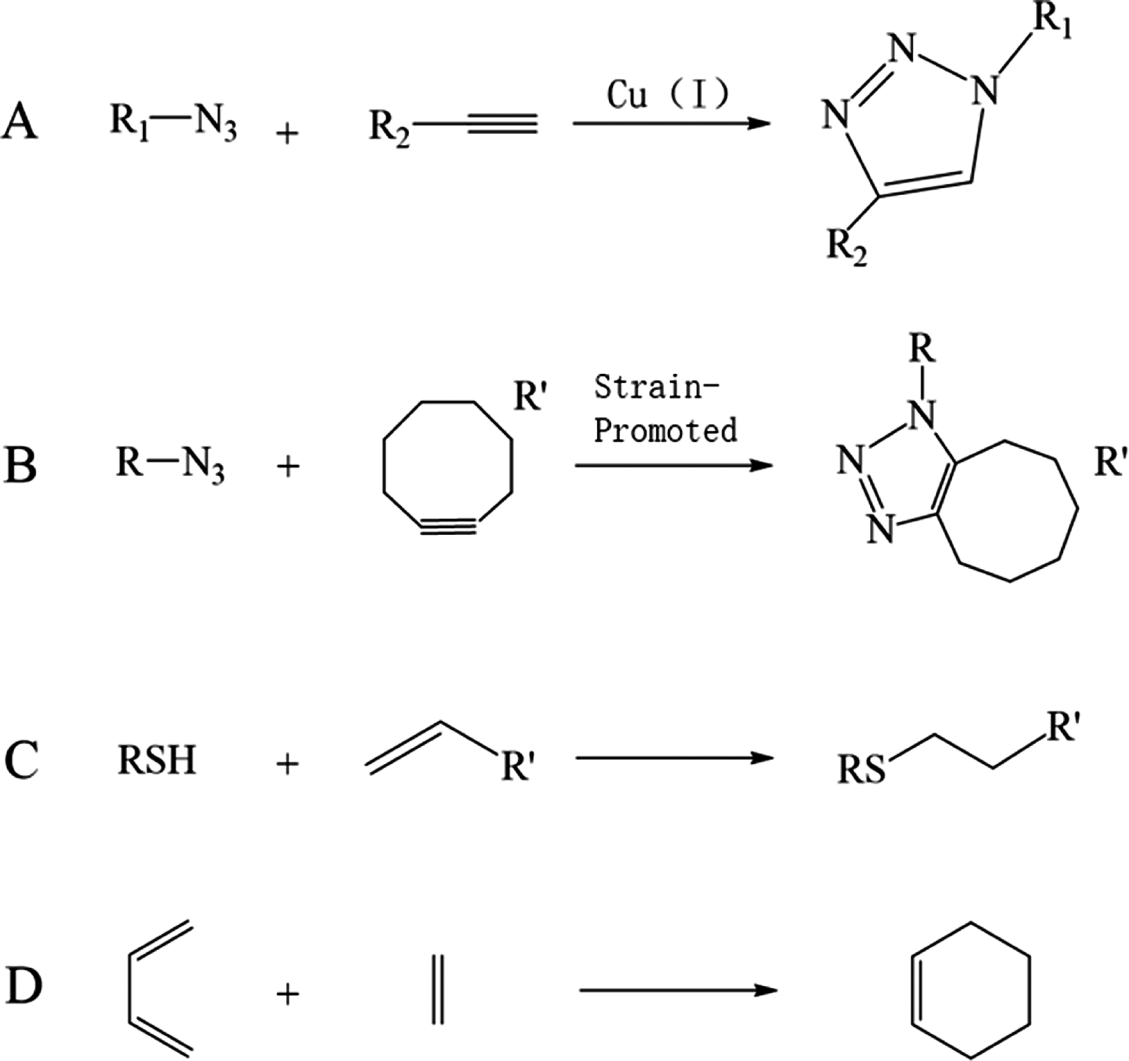
(A) Schematic diagram of CuAAC reaction. (B) Schematic diagram of the SPAAC reaction. (C) Schematic diagram of the thiol–ene reaction. (D) Schematic diagram of the Diels–Alder reaction.

The CuAAC reaction is a modified Huisgen 1,3-dipolar cycloaddition in which azide groups and alkynes are catalyzed by copper ions to generate 1,2,3-triazoles^32^ (Figure 1A). In the absence of a copper ion catalyst, this reaction is slow and nonselective, and the resulting products are 1,4- and 1,5-substituted isomers. When copper ions are introduced, the rate of the reaction is increased by 7 orders of magnitude,^33^ and selectivity generates only the 1,4-disubstituted isomer product.^34^ This heterocycle has some unique properties, such as oxidation resistance under acidic conditions and chemical inertness to hydrolysis,^35^ and can also participate in the formation of hydrogen bonds. Although 1,2,3-triazoles are not naturally formed, their biological properties have made them popular in a variety of fields.^36^ Due to the advantages of the CuAAC reaction, it is an efficient method for the preparation of hydrogels and is frequently used to design hydrogel networks.^37^ It has been used for many other polymeric materials for biochemistry and drug synthesis.

### 2.2 Strain-Promoted Azide–Alkyne Cycloaddition (SPAAC) Reaction

The traditional CuAAC reaction is the most commonly used “click” reaction, but since copper ions are used as the catalyst, biological toxicity is a major drawback. To address the limitations imposed by copper ions, Bertozzi’s group developed a reaction that used cyclooctane to react with azide groups to produce aromatic triazoles.^38^ This method requires no catalyst and proceeds at room temperature, known as copper-free “click” chemistry”^18^ (Figure 1B). In the absence of a catalyst, the reaction kinetics mainly depend on the structure of cyclooctane. To continuously adjust the reaction kinetics of this reaction, many cyclooctane derivatives have also been developed, such as bicyclononynes and difluorinated cyclooctyne (DIFO).^39,40^ Compared with the CuAAC reaction, this reaction does not greatly reduce the reaction rate and also has a high chemical selectivity and biocompatibility.^41^ This shows that this reaction has good potential and applicability in the field of biomedicine.

### 2.3 Thiol–Ene Reaction

Thiols are commonly used functional groups in cross-linking reactions and are easily obtained from the amino acid cysteine and can undergo “click” reactions with various functional groups. Here, an important reaction between thiol and alkene is introduced.^42^ Under the action of light or thermal initiators, thiol groups can react with alkenes to form thioethers^43^ (Figure 1C). The reaction has high selectivity and can be carried out in water (and is unaffected by water), and the reaction yield is almost 100%, which gives it good applications for hydrogels. Because it is a light-guided reaction, the reaction has good spatial control. By controlling the time, place, speed, etc. of the reaction by light,^44^ we can modify the internal spacing of some gels, which is uniquely attractive for hydrogels.^45^ Although most of the reaction is initiated using ultraviolet light, which has adverse effects on organisms, it can be made biocompatible by controlling the wavelength of light and the dose.^46^ So far, the thiol–ene reaction has been applied for medicinal chemistry, biomedicine, and polymeric materials,^47^ but there is also great room for development in the future.

### 2.4 Diels–Alder (DA) Reaction and IEDDA Reaction

The DA reaction is a cycloaddition reaction between an electron-rich diene and an electron-poor diene to form a six-membered ring^48^ (Figure 1D). This reaction can be carried out without a catalyst or coupling agent, is highly selective, and does not produce byproducts. Due to its hydrophobicity, the reaction rate is very fast in the presence of water.^18^ This reaction has been developed for some time and is most widely used in the maleimide–furan reaction.^49^ It is often used in the field of hydrogels such as tissue regeneration and cell encapsulation,^50^ but there are also applications in other fields. By improving this reaction, in 2008, Fox and his team reported the cycloaddition of a tetrazine and a strained alkene, called the IEDDA reaction.^51^ Compared with the traditional DA reaction, the IEDDA reaction has a faster reaction rate and better irreversibility.^52^ The tetrazine group is usually modified with additional electron-withdrawing groups to improve the stability of the reaction.^53^ This reaction also plays an important role in “click” chemistry. Among the biocompatible “click” reactions, it has the fastest rate and is also the first reaction to be used in clinical experiments.^54^

### 2.5 Nucleic Acid “Click” Chemistry

**Figure 2:**
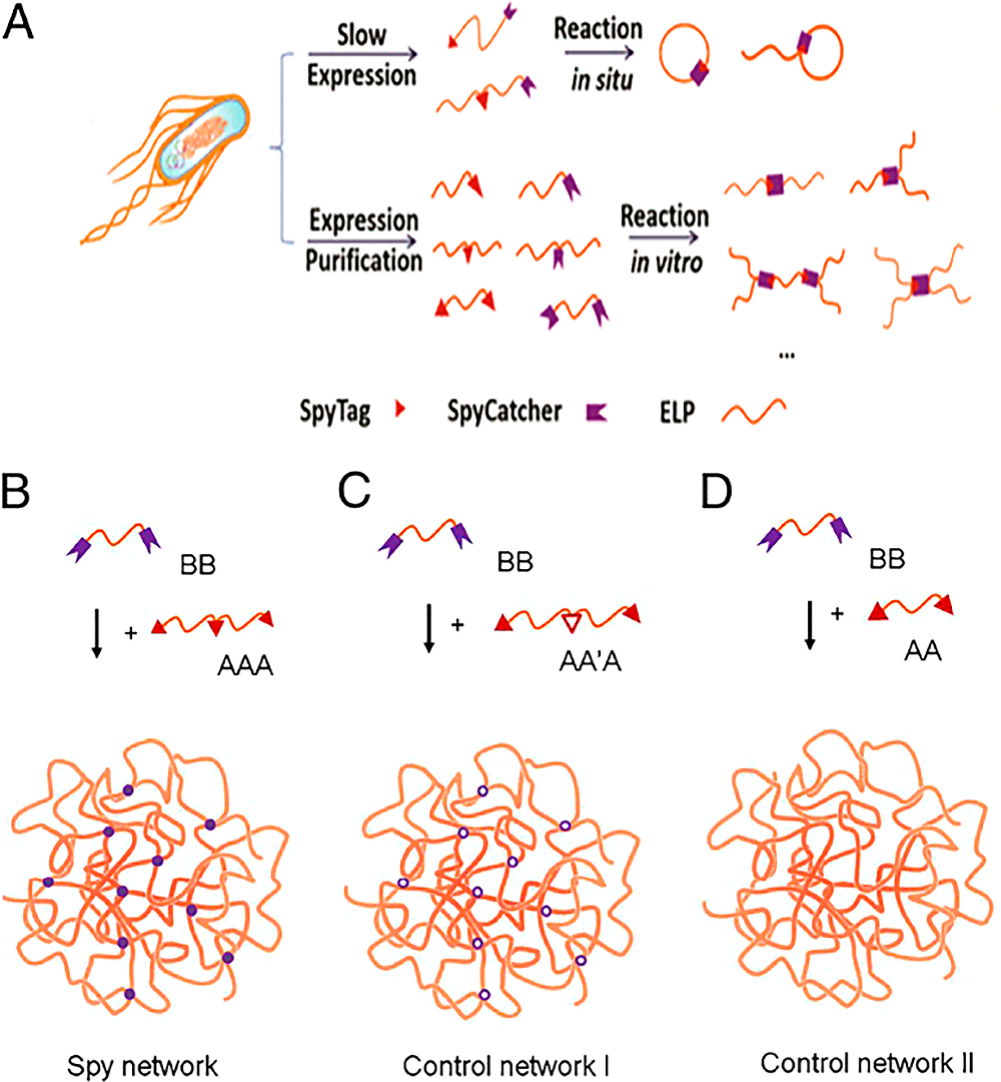
(A) SpyTag short polypeptides bind to their chaperone SpyCatcher to form proteins of various shapes under physiological conditions. Image reproduced from ref (56). Copyright 2013 American Chemical Society. (B–D) Schematic diagrams of different types of protein precursors forming hydrogels. Image used with permission from ref (59). Copyright 2014 Proceedings of the National Academy of Sciences.

The base pairing reaction in the DNA chain has the same advantages as other “click” reactions, including a fast rate, extremely high selectivity for base pairing, and no byproducts. Compared with other “click” reactions, the advantages of the nucleic acid “click” reaction are more prominent. Therefore, this review compares it with other “click” chemistry reactions to reasonably expand the concept of “click” chemistry.^55^ With improvements in biotechnology, Zhang and colleagues used genetic coding to control protein synthesis to produce desired structures, such as circles and stars, so that this reaction can be applied to prepare protein materials. The reaction had a fast rate and specificity.^56^ This reaction, known as the CECCs reaction, surpassed the CuAAC reaction in terms of kinetics.^57^ Coupled with protein engineering tools, this reaction extended “click” chemistry to the protein domain (Figure 2). Due to the particularity of the raw materials for this reaction, it is favored in many fields, such as the preparation of protein hydrogels and nucleic acid hydrogels.^58^ With the continuous development of biology, this reaction will see more applications in the future.

## 3 Application of “Click” Chemistry in the Field of Biomedical Hydrogels

Hydrogels have been widely used in tissue regeneration, cell culture, drug delivery, etc.^60^ “Click” chemistry plays an important role in the preparation of hydrogels. In this section, we review the applications of “click” chemistry-prepared hydrogels for drug delivery, cell culture, tissue repair, biosensors, and 3D bioprinting applications.

### 3.1 Drug Delivery

**Figure 3:**
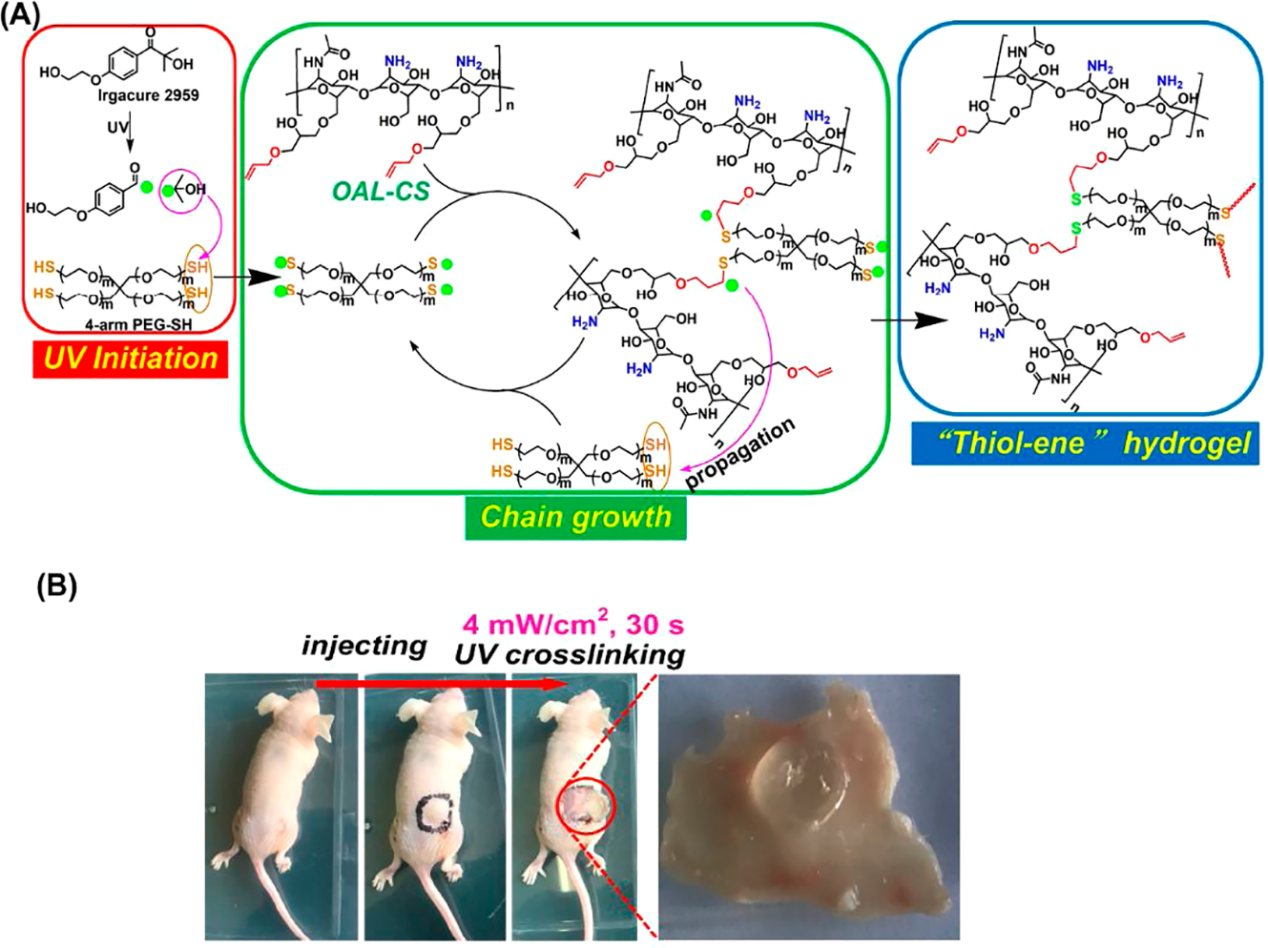
(A) Reaction mechanism of “click” chemistry, which can rapidly form hydrogels. (B) Formation of in situ hydrogels in mice takes only 30 s. Image reproduced with permission from ref (67). Copyright 2020 Elsevier.

Hydrogels have promising applications in the field of drug delivery.^61^ Their porosity has unique advantages^62^ and can controllably transport, protect, and release drugs, which have great advantages compared with traditional drug delivery materials.^63^ Due to the particularity of the drug delivery environment, the raw materials for synthesizing hydrogels generally include polysaccharides and polypeptides, among which abundant amino acid hydroxyl groups provide a broad platform for “click” chemistry. This makes “click” chemistry very valuable to this field.^64^ For example, the “thiol–ene” “click” reaction triggered by UV light results in a more uniform hydrogel network, lower biotoxicity, and less free radical generation than conventional chemical reactions.^65,66^ In 2021, Ding and colleagues used the “thiol–ene” reaction with modifiable chitosan to create pH-responsive hydrogels for drug delivery.^67^ Originally, it was difficult for chitosan to display pH-responsive behavior because its amino groups easily reacted with other groups.^68^ The authors used “click” chemistry to modify the surface of chitosan to protect the amino groups and retain the pH responsiveness brought by the amino groups. The hydrogel could be cross-linked within 30 s (Figure 3), which shows the practical value brought by “click” chemistry.

DNA hydrogels have very good biocompatibility, can be loaded on other nucleic acids, and are perfectly compatible with DNA drugs.^69^ They have an irreplaceable position in the field of drug delivery. The aforementioned nucleic acid “click” chemistry has played a key role in the synthesis of DNA hydrogels and has been widely recognized. For example, Zhang and his team used nucleic acid “click” chemistry to develop a DNA hydrogel that could be combined with the DNA drug doxorubicin and demonstrated that the drug worked better in hydrogels.^70^ The hydrogel showed the accumulation of cancer cells in vivo, and the detection efficiency was 3 times higher than that of conventional methods. This provided a powerful tool to detect the growth of cancer cells over time. In 2020, Yang et al. synthesized a protein-based hydrogel using a four-arm star-shaped protein.^57^ They used the “click” reaction to achieve a gel–sol transition under light induction, achieving the controlled release of drugs in the human body.

### 3.2 3D Cell Culture

The environment in which cells live is generally a dynamic 3D environment, and various factors jointly determine the growth process of cells.^71^ In the past, cell cultures were typically performed in 2D environments, which led to the failure of many experiments.^72^ Therefore, many scientists developed hydrogels for cell culture, which required cross-linking under physiological pH and temperature. The reactions must be fast and biocompatible, making “click” chemistry the best choice.^73^ DeForest and colleagues published an article in which they reacted four-arm poly(ethylene glycol) (PEG) tetraazide and bis(DIFO3)-difunctionalized polypeptide using a copper-free “click” reaction to rapidly generate cell culture hydrogels.^74^ Relying on the excellent biocompatibility of “click” chemistry, they directly encapsulated the cells in the hydrogel, making the cell growth environment more similar to an actual physiological environment. Through the thiol–ene “click” reaction, they photopatterned the hydrogel surface to detect the growth state of cells and promote cellular functions^75,76^ In 2019, Baker and colleagues designed a “click” hydrogel using hyaluronic acid (HA) to culture breast cancer cells.^76^ Compared with traditional 2D cultures, the hydrogel detected more genomes, and inhibitory drugs were more effective using this culture method. This confirmed the superiority of “click” chemistry-designed hydrogels for cell cultures.

### 3.3 Tissue Repair

Regenerative medicine is very important in biomedical fields.^77^ Biomaterials used for tissue regeneration are generally hydrogels and have high requirements, such as injectability, controlled release, biocompatibility, and degradability.^49^ A general chemical cross-linking method is difficult to achieve, and researchers have often chosen “click” chemistry for designing tissue regeneration hydrogels. Damage to cartilage tissue is irreversible and cannot rely on the human body to repair itself.^78^ Generally, bone tissue cells are quickly cultured, and after cells proliferate, they are implanted into damaged joints to complete tissue repair.^79^ When Erlane and his team used hydrogels to repair bone tissue, they found that the lack of oxygen molecules in the hydrogels affected the division of bone cells. Traditional oxygen-producing materials lacked continuous and controllable supply and were also toxic to cells. Therefore, “click” chemistry was used to repair bone tissue.^80^ They used the inverse electron demand Diels–Alder (IEDDA) “click” reaction to load oxygen-producing particles on the hydrogel, which provided a continuous supply of oxygen. The hydrogel was in situ cross-linked using the “click” reaction and possessed tunable mechanical properties and promoted bone tissue repair.

In 2018, Qu and his team reported a hydrogel for wound repair using chitosan and PF127-aldehydes and cross-linked them via a “click” reaction,^81^ which had the characteristics of hemostasis, antibacterial, self-healing, and adhesion. Compared with ordinary commercial dressings, the healing performance was more than doubled. Liu and co-workers introduced polydopamine (PDA)-decorated nHA (PHA) into sodium alginate and gelatin and used “click” chemistry to cross-link it to form a bone-repair hydrogel.^82^ The hydrogel achieved the in situ cross-linking at the bone injury site through “click” chemistry. It has been proven experimentally that hydrogels promote the repair of bone tissue, which solved the problem of filling after bone injury and avoided concurrent inflammation caused by surgery. Ocando and colleagues used alginate and alginate/Mg-doped hydroxyapatite (MgHAp) for cross-linking by “click” chemistry.^83^ The designed hydrogels were used as bioscaffolds that mimicked the porous structure of the bone tissue and provided space for bone cells to attach and proliferate, thus promoting bone tissue repair. These recent studies demonstrate that “click” chemistry has prominent applications in tissue repair hydrogels.

### 3.4 Biological Sensors

**Figure 4:**
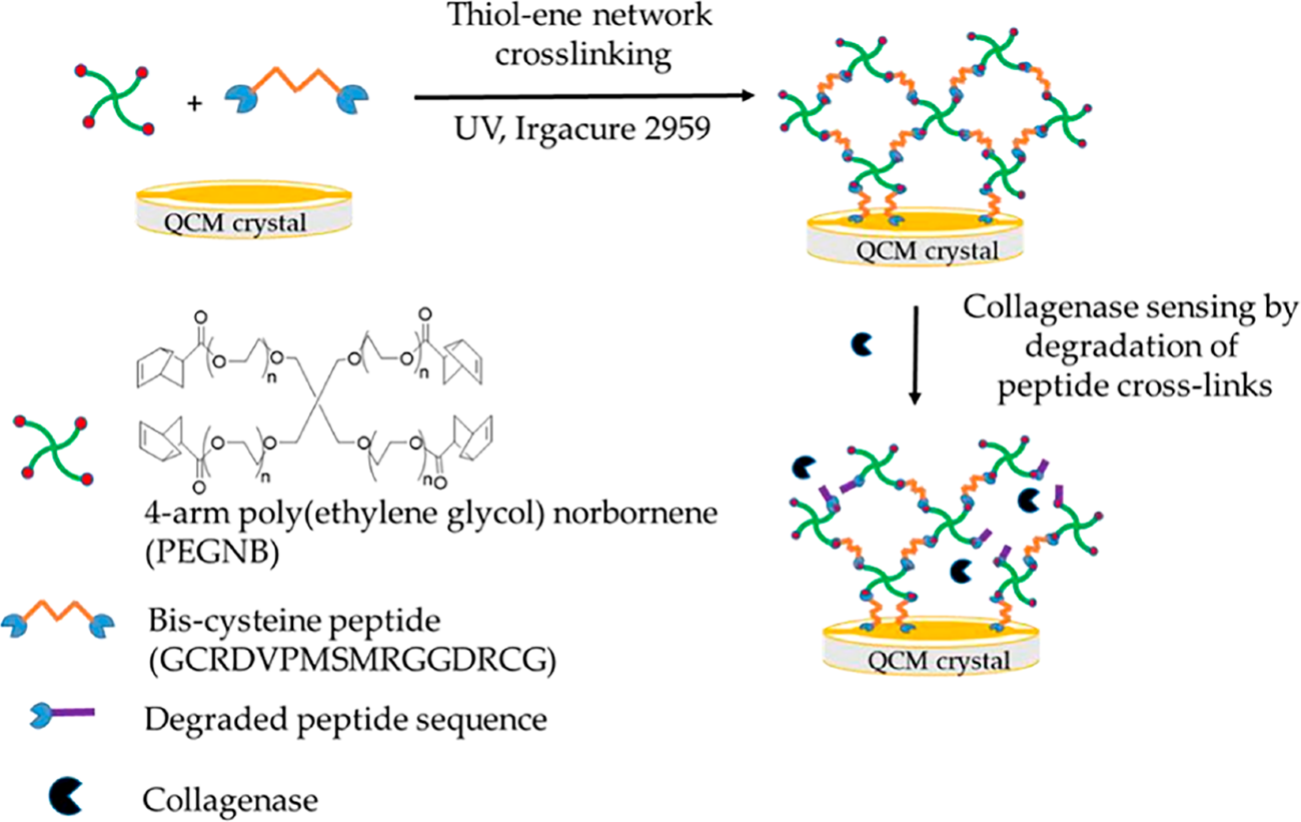
Schematic diagram of the synthesis of a hydrogel by “click” reaction and the schematic diagram of collagenase sensor. Image reproduced with permission from ref (87). Copyright 2019 MDPI.

Hydrogels are elastic soft materials that are suitable for wearable applications and as electronic skins and biosensors in medicine.^84^ In the past, silicon nanomaterials were often used, but they had shortcomings such as opacity, poor stretchability, and low temperature responsiveness.^85^ Now hydrogel materials have overcome these shortcomings, and the widespread use of “click” chemistry has allowed the field to be rapidly developed. Wang and co-workers layer-by-layer synthesized a hydrogel via an in situ Schiff base reaction composed of oxidized dextran (PO-Dex), chitosan, and glucose oxidase (GOD).^86^ Touching the hydrogel film to glucose caused a pH change, as the pH-responsiveness of the “click” reaction caused the film to swell. This changed the optical properties of the film, thereby monitoring the glucose concentration. The sensor’s response to the glucose concentration was reversible within a certain range, and it could continuously monitor the glucose concentration. The hydrogel film was very thin, so information was transmitted quickly, allowing it to monitor the blood glucose concentration in real-time. Ahmad designed a hydrogel film for collagenase monitoring using the thiol–ene reaction.^87^ The operating principle involved monitoring the degradation of the hydrogel by a quartz crystal microbalance (QCM) to detect collagenase (Figure 4). The device could be used to conveniently monitor osteoarthritis, and it had a nursing effect in the later stages of treatment.

### 3.5 3D Bioprinting

**Figure 5:**
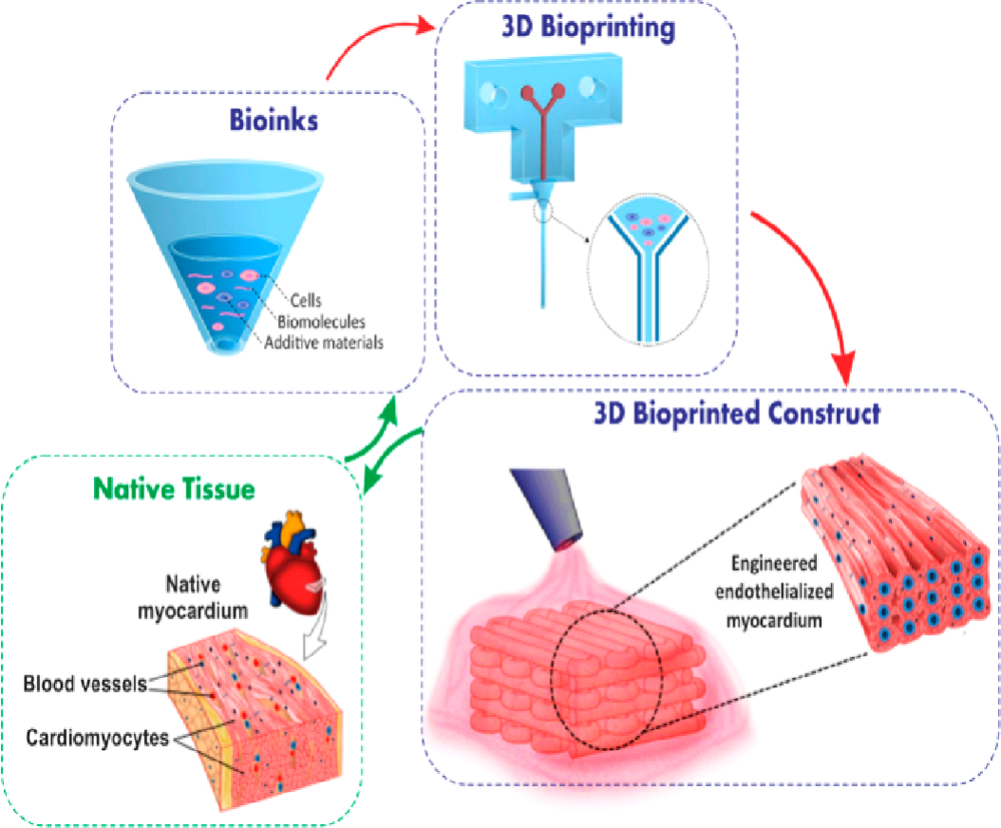
Schematic diagram of the design of the biological structure and its fabrication procedure. Image reproduced with permission from ref (90) (copyright 2019 Elsevier) and ref (95) (copyright 2016 Elsevier).

3D bioprinting is an emerging technology with great potential for biological applications.^88,89^ The difficulty of this technology is not the printing itself but the need to design suitable hydrogel materials for 3D printing to better simulate the structure and function of native tissues^90^ (Figure 5). These hydrogel materials must meet many requirements, such as good mechanical properties, biocompatibility, and fast cross-linking.^91^ To construct hydrogel materials for 3D printing, fast cross-linking under mild conditions and no biotoxicity are required. “Click” chemistry is the most practical approach,^92^ and many researchers have developed hydrogel inks for 3D printing using “click” chemistry. Jeon and colleagues used oxidized and methacrylated alginate (OMA) to assemble microgels that could be loaded with cells.^93^ Then they performed secondary cross-linking through a “click” reaction under UV irradiation to form 3D structured hydrogels. During this process, the required 3D structure could be designed according to the guidance of ultraviolet light. OMA well maintained the viability of internal cells and could be stored for a long time when frozen, allowing it to be applied as needed, providing a very practical tool for the subsequent fabrication of 3D structures. Similarly, many researchers have used alginate substances through thiol–ene “click” chemistry to premix the bioinks and then expose them to UV light during extrusion. Then cross-linking was performed using rapid “click” chemistry to construct 3D models.^19^ In 2017, Stichler and colleagues designed and developed a bioink for 3D printing by thiol–ene “click” chemistry.^94^ They verified the biotoxicity of the hydrogel with human bone-marrow-derived mesenchymal stem cells (hBMSCs) and used “click” chemistry to subsequently introduce hyaluronic acid to adjust the rheological properties of the hydrogel. They printed 20 layered structures, which improved the poor mechanical properties of most hydrogel materials.

## 4 Other Applications of “Click” Chemistry

### 4.1 Targeted Delivery of Drugs

**Figure 6:**
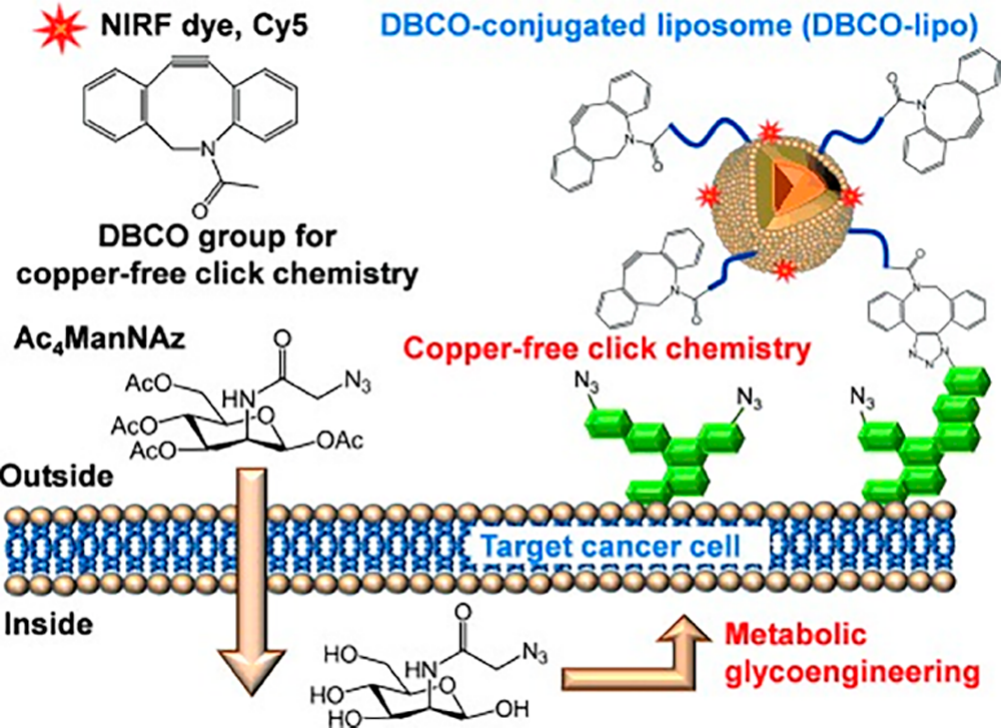
Schematic illustration of the in vivo tumor-targeting strategy of bioorthogonal copper-free “click” chemistry nanoparticles. Image reproduced with permission from ref (96). Copyright 2012 Wiley.

Drug delivery is an important medical method in modern medicine, and it is necessary to ensure a high efficiency, specificity, and bioorthogonality of the processes used to transport drugs to designated locations. These points cater to the characteristics of “click” chemistry. Combined with metabolic engineering, functional groups can be introduced at specific sites, and “click” reactions occur with special groups introduced on drugs. Its extremely high specific recognition and reaction efficiency provides an efficient drug delivery method. In 2012, Koo’s team injected intratumoral injections of tetraacetylated *N*-azidoacetyl-d-mannosamine (Ac4ManNAz), an unnatural substance with an azide group. Then alkynyl-modified liposomes were injected to perform a “click” reaction in mice, and it was found that many liposomes were bound to the surface of the cancer cells at a very fast rate^96^ (Figure 6). It is clear that by using “click” chemistry, drugs can be targeted to cancer cells and bind to receptors with a high efficiency. In 2016, Man’s team developed a cathepsin-specific metabolic precursor that could easily generate azide-containing receptors outside tumor cells.^97^ In the report, they successfully used a bioorthogonal reaction to bind to the receptor, which greatly enhanced the practicality of “click” chemistry in drug delivery and showed that this reaction has a great potential in the future of drug delivery. In Lee’s 2014 report, to address the tumor-targeting strategy of nanoparticles, metabolic engineering was first used to introduce azide groups on the surface of tumor cells. Then through the copper-free “click” reaction in vivo, it enhanced the targeting ability of nanoparticles to cancer cells.^98^ Compared with traditional targeting strategies, this method was simpler to operate, had more sites on cancer cells, and had a higher binding rate of nanoparticles. This reflects the application prospects of “click” chemistry in this field.

### 4.2 Cell Labeling

**Figure 7:**
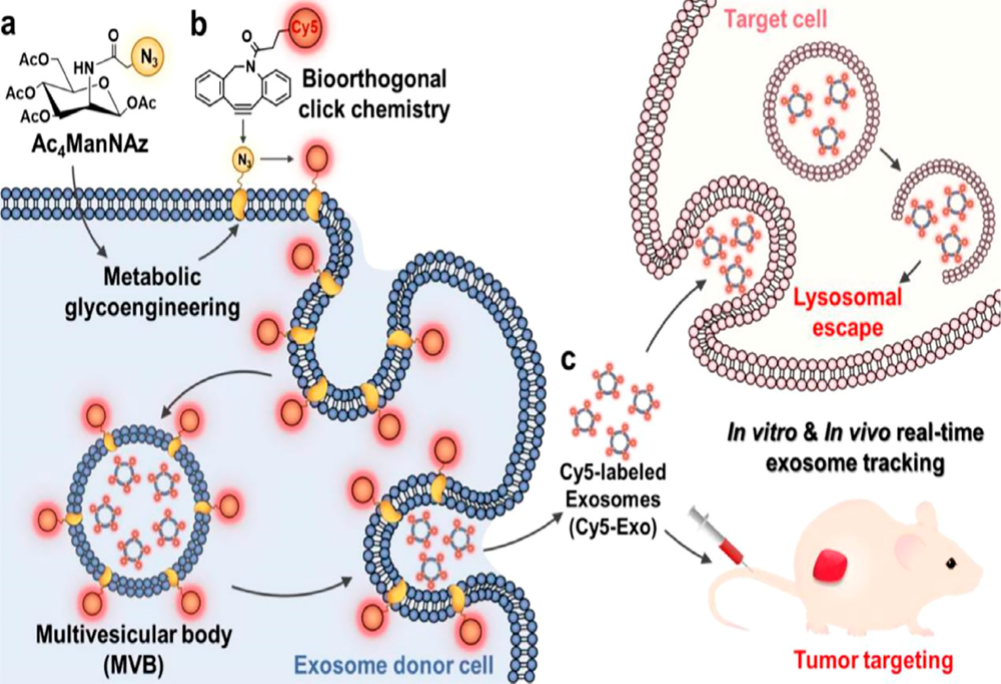
Schematic illustration of in situ one-step bioorthogonal “click” chemistry of metabolite-treated cancer cell exosomes. Image reproduced from ref (99). Copyright 2020 American Chemical Society.

Cell labeling is an effective means in biomedicine that is often used for detection, treatment, and evaluation to predict disease conditions. The current traditional cell labeling method is generally completed by methods such as isotope labeling. Traditional methods can easily label cells, but they have obvious shortcomings. The disadvantages of traditional labeling methods include a low efficiency, short effective labeling time, and signals that easily change when cells are active. Labeling by “click” chemistry has a higher reaction efficiency, and the labeling duration is long and does not easily change.^99^ The most commonly used CuAAC reaction in “click” chemistry was initially inseparable from the catalysis of copper ions and was not suitable for biological reactions. However, with continuous development, various copper-free CuAAC reactions have appeared and can be used for cell labeling without affecting the biological activity of cells.^100^ Therefore, a bioorthogonal “click” reaction can be used to label exosomes in one step in situ, providing high yields of fluorescently labeled exosomes without changing the intrinsic properties of natural exosomes (Figure 7). In 2016, Lee reported that his team used metabolic engineering to introduce azide groups on stem cells and used copper-free “click” chemistry to fluorescently label the surface of stem cells.^101^ The stem cells were transplanted into mice with hindlimb ischemia, and the therapeutic effect of stem cells on the target disease was monitored by an optical imaging system. The obtained images were clearer and more stable than those obtained using traditional labeling methods. In 2016, Yoon also used Ac4ManNAz to introduce azides on the surface of chondrocytes and then reacted them with an alkynyl group labeled with a near-infrared fluorescent dye to label chondrocytes.^102^ By varying the amount of the starting material, the team easily changed the number of azide groups and easily controlled the concentration of labeled cells. Yoon and his colleagues implanted labeled chondrocytes into mice and observed the formation of mouse cartilage tissue for 4 weeks. The effective labeling time was more than twice that of the conventional method, proving that the “click” reaction did not affect the subsequent cartilage tissue formation. These two examples illustrate the universality of this “click” reaction, and it is believed that, in the future, more cell labeling will be accomplished using “click” chemistry.

### 4.3 Drug Synthesis

“Click” chemistry has a high reaction yield, almost no byproducts, and a fast reaction rate. These advantages are undoubtedly the dream of drug preparation. Therefore, “click” chemistry has attracted much attention in the field of drug synthesis.^103^ Because the original “click” reaction relies on copper ions that are toxic to organisms, it has not been frequently used in the field of medicine. However, with the development of “click” chemistry and the introduction of various copper-free “click” chemistry reactions, “click” chemistry has become very popular in the field of biomedicine, as well as for drug synthesis. “Click” chemistry provides an easy method to synthesize 1,2,3-triazoles, which have good pharmacological effects and have been developed and applied to the synthesis of various antiviral agents, antibacterial agents, and anticancer agents.^104^ In 2016, Kant and his colleagues synthesized 25 1,2,3-triazole derivatives using “click” chemistry and tested their antibacterial properties against Gram-positive bacteria, Gram-negative bacteria, and other strains.^105^ Similarly, in 2018, Karypidou’s research team used “click” chemistry to synthesize a series of new 1,2,3-triazole derivatives and used them to evaluate the coronavirus.^106^ The results showed that they had strong antivirulence against the coronavirus, indicating that they were promising for the prevention and treatment of the coronavirus.

### 4.4 Surface Modification

**Figure 8:**
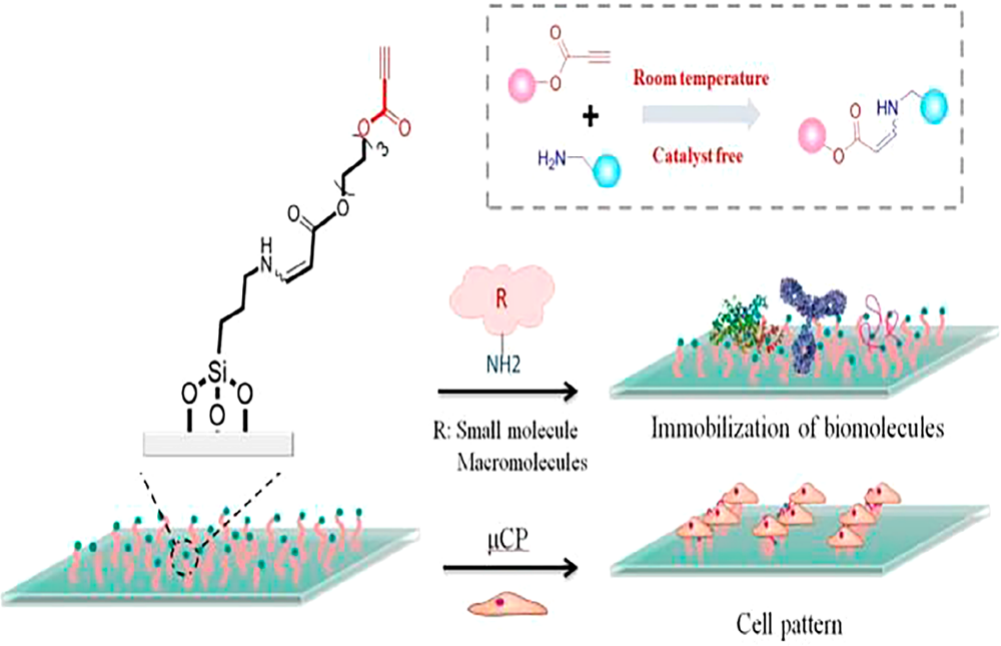
Schematic illustration of the rapid surface immobilization of native bioconjugates via spontaneous “click” reactions. Image reproduced with permission from ref (109). Copyright 2020 The Royal Society of Chemistry.

Surface immobilization and modification are very useful for biomedicine and provide a good platform for biochemical reactions.^107^ It has important significance for drug screening and tissue engineering.^108^ Traditional modification methods are inefficient, and their reaction processes are very complicated, which limits the progress of surface modification work. “Click” chemistry reactions have a high efficiency and are basically complete in one step, using a simple reaction process. These advantages are very helpful for surface modification. In 2020, Zhang and colleagues developed a surface immobilization technique that utilized an aminoalkyne “click” reaction to immobilize bovine serum albumin on the surface of a glass slide within 30 min. This was several times faster than conventional immobilization methods^109^ (Figure 8). By capturing cells from solution, the bioactivity of the biomolecules immobilized by the “click” chemistry method remained more intact, and the number of immobilized proteins was significantly increased. The research group also used this method to draw patterns on the surface of seed cells, which proved that “click” chemistry has a good future for surface biofunctionalization. In surface drawing, photolithography a traditional method, but the process requires ultraviolet light and organic solvents, which are harmful to biological functions.^110^ In 2013, Arnold and his team introduced a method for surface drawing that did not require complex techniques or expensive conditions to orderly arrange biomolecules on surfaces.^111^ Using 1-aminomethylpyrene (AMP) and 5-azidofluorescein (AF) covalently bound to a polymer brush, the surface was mapped using the “click” reaction of azide and copper, followed by microscopy. The results showed that no cross-contamination occurred. This method can easily functionalize the surface and create different patterns on-demand, which can replace photolithography, electron beam, or ion beam. The development of “click” chemistry for surface modification has also improved the operability of future drug delivery methods and is expected to be a common means of future medicine.

## 5 Summary and Outlook

“Click” chemistry has attracted broad attention in the field of chemistry due to its unique advantages. Especially in recent years, it has been widely used to design polymeric hydrogels. In this article, we focus on several common “click” chemistry reactions and expand the concept of “click” chemistry to the field of nucleic acids. By introducing the application of “click” chemistry-prepared hydrogels in biomedical fields, the advantages and characteristics of “click” chemistry are further clarified. It is hoped that this review will provide a good toolbox for researchers engaged in hydrogel development. We also supplement the applications of “click” chemistry outside the field of hydrogels, such as drug synthesis, cell labeling, and the targeted delivery of drugs. This is of great help to our comprehensive understanding of “click” chemistry, and hydrogels have great potential for future applications in these fields, by reviewing these applications we hope to inspire the synthesis of hydrogels.

In general, “click” chemistry has excellent applications for the preparation of biomedical hydrogel materials, and more researchers are now considering the use of “click” reactions when designing hydrogels. The rapidity and efficiency of the “click” reaction help design injectable hydrogels. Because “click” reactions are a small group of reactions, they are simple and fast, and many scientists have applied them for the modification and functionalization of hydrogels. Most “click” chemistries also have good biocompatibility and can be used to prepare biomedical hydrogels, which is one of the reasons for the rapid development of biomedical hydrogels in recent years. Although “click” chemistry has made rapid progress in the field of biomedical hydrogels, there are still many problems to be solved. When a hydrogel prepared by “click” chemistry is used to replace human tissue, there is still a certain gap between the mechanical properties and human tissue. Currently, it is mainly solved by introducing a double network structure. Improving the performance of hydrogels by flexibly introducing special groups and structures and increasing the degree of cross-linking of hydrogels may be an important way to solve such problems. “Click” chemistry hydrogels are widely used in the field of biomedicine due to the biocompatibility of the reaction. Even so, because mainstream natural hydrogel materials are absolutely incompatible with the reaction, the reactants must be modified, resulting in products that may not comply with regulatory strategies. There are still only a few hydrogel products that can be directly applied in the clinic. This problem requires the continuous improvement of “click” chemistry and the accumulation of safety information about these reactions in the future.

Looking to the future, the development of modern medicine has an increasing demand for new hydrogel materials. “Click” chemistry can introduce innovative elements into hydrogels, such as optics, thermal effects, and magnetism, and these smart hydrogels will have a wide range of applications and prospects in new fields such as cancer treatment and artificial organs. There is also cell therapy, which promotes the combination of hydrogel and cells through “click” chemistry and regulates the biological environment of hydrogel to ensure the growth and reproduction of cells. This is an important idea for developing these materials in the future. For 3D bioprinting, the current technology is not mature enough, including the selection of polymerized monomers, cross-linking methods, and printing technology. “Click” chemistry can help adjust the gel time, printing ink viscosity, and control the degradability. With improvements in the technology of “click” chemistry in hydrogels and increasing demand for such hydrogels in the biomedical field, the future application prospects of “click” chemistry for biomedical hydrogels are worth looking forward to.
